# Visualisation of lenticulostriate arteries using contrast-enhanced time-of-flight magnetic resonance angiography at 7 Tesla

**DOI:** 10.1038/s41598-022-24832-z

**Published:** 2022-11-24

**Authors:** Christopher N. Osuafor, Catarina Rua, Andrew D. Mackinnon, Marco Egle, Philip Benjamin, Daniel J. Tozer, Christopher T. Rodgers, Hugh S. Markus

**Affiliations:** 1 Stroke Research Group, Department of Clinical Neurosciences, University of Cambridge, Cambridge, UK; 2 Department of Clinical Neurosciences, University of Cambridge, Cambridge, UK; 3 GB Invicro LLC, a Konica Minolta Company, London, UK; 4 Atkinson Morley Regional Neuroscience Centre, St George’s University Hospitals NHS Foundation Trust, London, UK; 5 Wolfson Brain Imaging Centre, Department of Clinical Neurosciences, University of Cambridge, Cambridge, UK

**Keywords:** Neuroscience, Neurology

## Abstract

7 Tesla-field-strength (7 T) Magnetic Resonance Imaging allows the small perforating arteries in the brain to be visualised, and this modality may allow visualisation of the arterial pathology in cerebral small vessel disease. Most studies have used standard Time-of-Flight (ToF) Magnetic Resonance Angiography (MRA). Whether the use of contrast enhancement improves perforating artery visualisation at 7 T remains unclear. In a prospective study, we compared standard ToF MRA with contrast-enhanced (CE) ToF MRA at 7 T for the visualisation of the lenticulostriate arteries (LSAs). Ten patients with symptomatic lacunar stroke were recruited (mean age, SD, 64 ± 9.9 years). Visualisation was assessed using a visual rating scale administered by two independent expert readers and length of the LSAs visible. Visualisation of the LSAs was improved with CE ToF MRA. The mean Visibility and Sharpness Score was higher for CE ToF MRA over standard ToF MRA (2.55 ± 0.64 vs. 1.75 ± 0.68; *P* = 0.0008). The mean length of LSA visualised was significantly longer with CE ToF MRA compared to standard ToF MRA (24.4 ± 4.5 vs. 21.9 ± 4.0 mm; *P* = 0.01). CE ToF MRA offers improved visualisation of the LSAs over standard ToF MRA. The addition of contrast may improve the ability to visualise cerebral small vessel disease arterial pathology.

## Introduction

Disease of the small cerebral perforating arteries, which supply the white matter and deep grey matter structures (cerebral small vessel disease, cSVD), causes a quarter of all strokes (lacunar strokes) and is the most common pathology underlying vascular cognitive impairment^1^. Until recently, it has not been possible to visualise the perforating arteries in humans using non-invasive techniques. More recently, high resolution 7 Tesla-field-strength (7 T) Magnetic Resonance Imaging (MRI) has allowed visualisation of perforating arteries because of the intrinsic increase in signal-to-noise ratio compared to lower field strengths at 1.5 T or 3 T^2–4^. Studies have reported diffuse abnormalities in the perforating arteries in individuals with both stroke^5^ and stroke risk factors such as hypertension^6^, and have identified disruption of flow in individual perforating arteries in patients with lacunar infarcts^7^. 7 T MRI has the potential to provide important insights into the arterial pathology associated with lacunar stroke and cSVD, like increased tortuosity^5^ and occlusion of blood flow in individual perforating arteries^7^.

Many studies using 7 T MRI to date have used standard Time-of-Flight (ToF) Magnetic Resonance Angiography (MRA) without contrast^3–7^. Contrast-enhanced (CE) MRA is the collective name for several pulse sequences that acquire rapid sequential 3-dimensional (3D) imaging following injection of a bolus of a gadolinium-containing contrast agent^8^. There has been no systematic studies which we could find, evaluating whether the addition of a gadolinium based contrast agent improves visualisation of the small perforating arteries, typically between 200 and 500 µm in diameter. Arterial blood affected by contrast agent, will have shorter T_1_, thereby yielding higher signal than tissues not affected by the contrast agent^9^. CE MRA is widely used for visualisation of the larger arteries, including the extracerebral carotid and vertebral arteries for the visualisation of stenosis^10,11^. Studies have suggested that contrast administration provides increased signal to noise and improved visualisation compared to standard ToF MRA^2,12^, but it could also compromise visualisation by improving the visibility of veins, thereby increasing venous contamination. One 7 T study retrospectively analysed data from a mixed cohort of patients, and suggested contrast enhancement may increase the length of perforating arteries which can be visualised^13^, but no systematic analysis of its benefits has been performed.

In this prospective study of patients with lacunar stroke secondary to cSVD, we compared standard ToF MRA and ToF MRA with the addition of a contrast agent (CE ToF MRA) at 7 T within clinically acceptable acquisition times. We assessed: (1) whether there was any improvement in radiological visualisation of the lenticulostriate arteries (LSAs); (2) the length and number of the individual LSAs that could be identified, and (3) the signal within the LSAs to noise ratio. We hypothesized that CE ToF MRA improves the delineation of LSAs compared to standard ToF MRA at 7 T. To determine its utility in patients with cSVD, in whom perforating artery visualisation may be inferior to normal controls, we performed the comparison in individuals with symptomatic lacunar strokes.

## Methods

### Study population

Inclusion criteria was a clinical lacunar syndrome with an anatomically corresponding lacunar infarct on brain MRI. Participants with any other potential cause of stroke other than cSVD (including large artery disease causing a stenosis > 50%, or a cardio-embolic source), or with any other potential cause of white matter disease (such as multiple sclerosis) were excluded. Additionally, subjects with a contraindication for 7 T MRI (for example, claustrophobia, specific metal objects in or on the body, pregnancy, known allergy to gadolinium-containing contrast agent, or impaired renal function with estimated glomerular filtration rate of less than 59 mL/min/1.73 m^2^) were excluded. This study was approved by the Institutional Review Board of East of England–Cambridge Central Research Ethics Committee (REC Ref: 19/EE/0219). Written informed consent was obtained from all participants. Participants were recruited between February 2020 and November 2020.

### MR imaging protocol

A 3D ToF MRA technique was performed on a whole-body human 7 T MR system (7 T Terra, Siemens Healthineers, Erlangen, Germany) equipped with a 32-channel receive coil (Nova Medical, Wilmington, Massachusetts). Before image acquisition, a 20-gauge intravenous cannula was placed in the antecubital vein for contrast injection. Firstly, ToF MRA pulse sequences were acquired; this was followed by manual intravenous administration of 0.1 mmol/kg of a gadolinium-based contrast agent [Gadobutrol, Gadovist®, Bayer PLC, Reading, UK)^14^ and 10 millilitres of 0.9% sodium chloride flush and finally, acquisition of post contrast MRA pulse sequences. The CE ToF MRA pulse sequences were acquired two minutes after contrast injection in nine participants; in one participant, pulse sequences were acquired at the same time of contrast injection. No changes were made to the sequence parameters after contrast administration. The following protocol was used for both the pre and post contrast sequences: Field of view (FOV) 200 × 156.3 mm^2^, Voxel Size 0.24 × 0.24 × 0.32 mm^3^, repetition time (TR) of 13 ms, echo time (TE) of 5.1 ms and flip angle (FA) of 20 degrees. Two slabs were used with 80 slices per slab to shorten the time of flight of inflowing blood and, thus increase the blood signal. To accelerate image acquisition, a partially parallel acquisition mode, GeneRalized Autocalibrating Partially Parallel Acquisition (GRAPPA) with an acceleration factor of two was used. Acquisition time (TA) was 9 min and 53 s. Standard pulse sequences which include T_1_-weighted sequence, T_2_-weighted sequence, T_2_-weighted fluid-attenuated inversion recovery (FLAIR) and diffusion-weighted sequences, were also obtained for the purpose of identifying radiological markers of cSVD like number of lacunes/lacunar infarcts, grade of white matter hyperintensities, number of microbleeds and any abnormal brain lesions.

### Postprocessing and data analysis

All images were exported to an offline station equipped with Syngo® (Siemens) and Weasis® medical image viewer software for image processing. Multiplanar Reconstructions (MPR) were performed in coronal, sagittal and axial planes. Maximum Intensity Projections (MIPs) were reconstructed in the coronal and axial (slab thickness 15 mm) directions. The MIPs were focussed on the main trunk of the middle cerebral artery (MCA) and anterior cerebral artery (ACA) to centre the LSAs. 3D-volume rendered images were used to ensure anatomical landmarks of the MCA and ACA. LSAs arising from the MCA (lateral LSAs) and horizontal part of the ACA (medial LSAs) were identified. Standard pulse sequences were for used for standard reporting to assess for lacunar infarcts, white matter hyperintensities, enlarged perivascular spaces and microbleeds.

### Comparison of standard ToF MRA and CE TOF MRA images

#### Visual rating scale

A qualitative visual rating scale was used to compare standard ToF MRA with CE ToF MRA using a scale, modified from work done previously on vessel imaging^15,16^. Rating was performed independently by two raters (one clinical research fellow, C.N.O. and one consultant neuroradiologist, A.D.M.) who were blinded to each other’s rating, with both images placed side by side. When scores differed significantly (> 2 point difference) between the 2 raters, the images were re-evaluated together, and the score determined by consensus. Overall visibility and sharpness of the LSAs, presence of artefacts and extent of venous contamination were rated.

i. Visibility and sharpness of the LSAs was assessed on a four-point rating scale (0, no LSA visualized; 1, poorly defined LSAs; 2, well defined LSA; 3, excellent definition of the LSA). This was performed on the coronal MIPs.
ii. Presence of artefacts (subject-dependent motion artefacts and flow artefacts around the main trunk of the MCA and the ACA) was assessed on a four-point rating scale (0, no artefact affecting visibility; 1, mild artefacts; 2, moderate artefacts; 3, severe artefacts). This was performed on the original images before MIP reconstruction.
iii. Extent of venous contamination of the image was assessed as cerebral veins and the dural venous sinuses (as well as arteries) were enhanced on the CE images. It was determined whether enhancement of the venous system (especially the basal cerebral veins and internal cerebral veins and their tributaries) affected the ability of the rater to visualise the origin or course of the LSAs. This rating was done on the axial MIP based on a four-point rating scale (0, no venous contamination affecting visibility; 1, mild venous contamination; 2, moderate venous contamination; 3, significant venous contamination).

#### Length and number of LSAs visualised

Length was measured (in millimetres) as the length of the most lateral LSA as a straight (through-space) linear distance from the origin of the LSA at the MCA or ACA, to its most distal visible part. These measurements were performed by one rater on the coronal MIP and was reported for all subjects (Supplementary Fig. 2). The number of visible LSAs originating directly from the MCA and the ACA and pointing towards the anterior perforated substance were counted. The vessels were counted from the most laterally located LSAs towards the most medially located LSAs as a total number of end branches, either as a single vessel (where one vessel exists without any branches) or as multiple vessels (where one vessel has more than one branch)^17^.

#### Signal to noise ratio

The conspicuity of the LSA relative to the background was analysed by calculating the Signal to Noise Ratio (SNR) of the LSA, SNR of brain and Contrast to Noise Ratio (CNR) of the LSA to the background brain tissue. This was also done by a single rater. SNR was defined as 0.695 x (SI/σ)^18^, where SI is signal intensity of the blood in the LSA, σ is the standard deviation of the noise determined in a signal-free, artefact-free background region, and 0.695 is the Rayleigh distribution correction factor to calculate the true SNR. The SI of the blood in the LSA was measured on the most prominent LSA on either side, as close as possible to its origin (from the MCA or ACA) using a region of interest (ROI) area of 0.2 mm^2^. SI of brain was measured using an ROI area of 20mm^2^ at an area of brain adjacent to the origin of the LSA. σ was measured on an ROI with area of 50mm^2^, drawn (in-air) at the posterior outer corner of the most inferior slice of the image. This was to avoid artefacts introduced into the background from the eyes (which are always in motion) in the anterior outer corners of the most superior slices. Finally, the CNR of the LSA to brain (CNR_LSA-BRAIN_) was defined as SNR_LSA_ minus SNR_BRAIN_^18^, where SNR_LSA_ is the SNR of the LSA and SNR_BRAIN_ is SNR of brain. For SI measurement and placement of ROIs, an axial plane on the original data was used as this gave the best view of the LSAs; this also enabled drawing on the same plane, both the background noise standard deviation ROI and the brain ROI on the anterior (adjacent) aspect of the MCA (Supplementary Fig. 1). When the LSAs were not visible on this plane, signal intensity was assessed on the coronal plane; in likewise fashion, the brain ROI was drawn adjacently.

### Statistical analysis

Statistical analysis was performed using R statistical software version 3.4.1^19^. Inter-rater variability between the two raters for the visual rating scale, was assessed with the Cohen’s weighted kappa using R package irr^20^. The strength of the inter-rater agreement was categorized as follows: ≤ 0, no agreement; 0.01–0.20, none to slight; 0.21–0.40, fair; 0.41– 0.60, moderate; 0.61–0.80, substantial; 0.81–1.00 almost perfect agreement. The mean qualitative difference between standard ToF MRA and CE TOF MRA across the two raters was analysed with a Wilcoxon signed-rank test and plotted using R package ggpubr^20^. The quantitative difference was also analysed using Wilcoxon signed-rank test for all measures except number of visible LSAs and Straight Length of LSAs where due to their parametric distribution, a paired t-test was employed. Statistical significance was defined as *P* < 0.05.

### Ethical approval

The study was conducted according to the guidelines of the Declaration of Helsinki and approved by the Institutional Review Board of East of England–Cambridge Central Research Ethics Committee (REC Ref: 19/EE/0219).

### Informed consent

Written informed consent was obtained from all participants in this study.

## Results

### Subjects

Ten participants (four males, six females) were included. Mean age was 64 ± 9.9 years (range 51–80 years). Demographic and clinical data of subjects are summarized in Table 1.

**Table 1:** Baseline demographics, vascular risk factors and radiological characteristics of participants.

Characteristic	Patients (*n* = 10)
Gender (female)	6 (60%)
Age at stroke onset, years	64 (51–80)
Ethnicity (White British)	9 (90%)
BMI, kg/m^2^ *	26.4 (25.6–30.8)
Modified Rankin score at consent	0 (0–2)
**Vascular risk factors**	
Hypertension	10 (100%)
Hyperlipidaemia	9 (90%)
Diabetes mellitus	1 (10%)
Smoking history (current or previous)	9 (90%)
Alcohol intake (≥ 14 units/week)	3 (30%)
Previous stroke/TIA	3 (30%)
**Number of lacunes/lacunar infarcts**	
1	5 (50%)
> 1	5 (50%)
**Fazekas grading of white matter hyperintensities**	
Grade 0	0
Grade 1	9 (90%)
Grade 2	0
Grade 3	1 (10%)
**Microbleeds**	
0	9 (90%)
1	1 (10%)
> 1	0

Categorical variables are expressed in counts and percentages; ordinal and continuous variables are expressed in median (range), except for age which was expressed as mean (range). BMI, Body Mass Index; Fazekas grading of White Matter Hyperintensities at periventricular white matter 0 = absent; 1 = caps or pencil-thin lining; 2 = smooth halo; 3 = irregular periventricular signal extending into the deep white matter; and at deep white matter: 0 = absent; 1 = punctate foci; 2 = beginning confluence; 3 = large confluent areas. *BMI not available in 2 patients.

### Visual rating scale

Inter-rater agreement for the qualitative analysis was moderate for Overall Visibility and Sharpness Score, fair to moderate for Presence of Artefact Score, and substantial to perfect for Extent of Venous Contamination Score as shown in Table 2. Since inter-rater agreement between both raters was moderate to perfect indicating good agreement, the mean scores for both raters were used for the qualitative comparison of the standard ToF MRA with the CE TOF MRA.

**Table 2:** Inter-rater agreement between raters for qualitative analysis.

Qualitative analysis scales	Pulse sequence	Percentage agreement between raters (%)	Weighted kappa	*P* value	Strength of agreement
Overall Visibility and Sharpness Score	Standard ToF MRA	70	0.595	0.01*	Moderate
	CE ToF MRA	70	0.531	0.04*	Moderate
Presence of Artefacts Score	Standard ToF MRA	60	0.375	0.09	Fair
	CE ToF MRA	60	0.444	0.04*	Moderate
Extent of Venous Contamination Score	Standard ToF MRA	100	NA	NA	Perfect^#^
	CE ToF MRA	90	0.615	0.04*	Substantial

Inter-rater agreement for the qualitative analysis was moderate for Overall Visibility and Sharpness Score, fair to moderate for Presence of Artefact Score, and substantial to perfect for Extent of Venous Contamination Score. NA = Not available as scores of zero (0) were not computed. ^#^Perfect agreement based on 100% agreement between the two raters. Strength of agreement categorised as ≤ 0, no agreement; 0.01–0.20, none to slight; 0.21–0.40, fair; 0.41– 0.60, moderate; 0.61–0.80, substantial; 0.81–1.00 almost perfect agreement. *Statistical significance reached (*P* < 0.05). ToF, Time-of-Flight; MRA, Magnetic Resonance Angiography; CE, Contrast-Enhanced.

**Figure 1:**
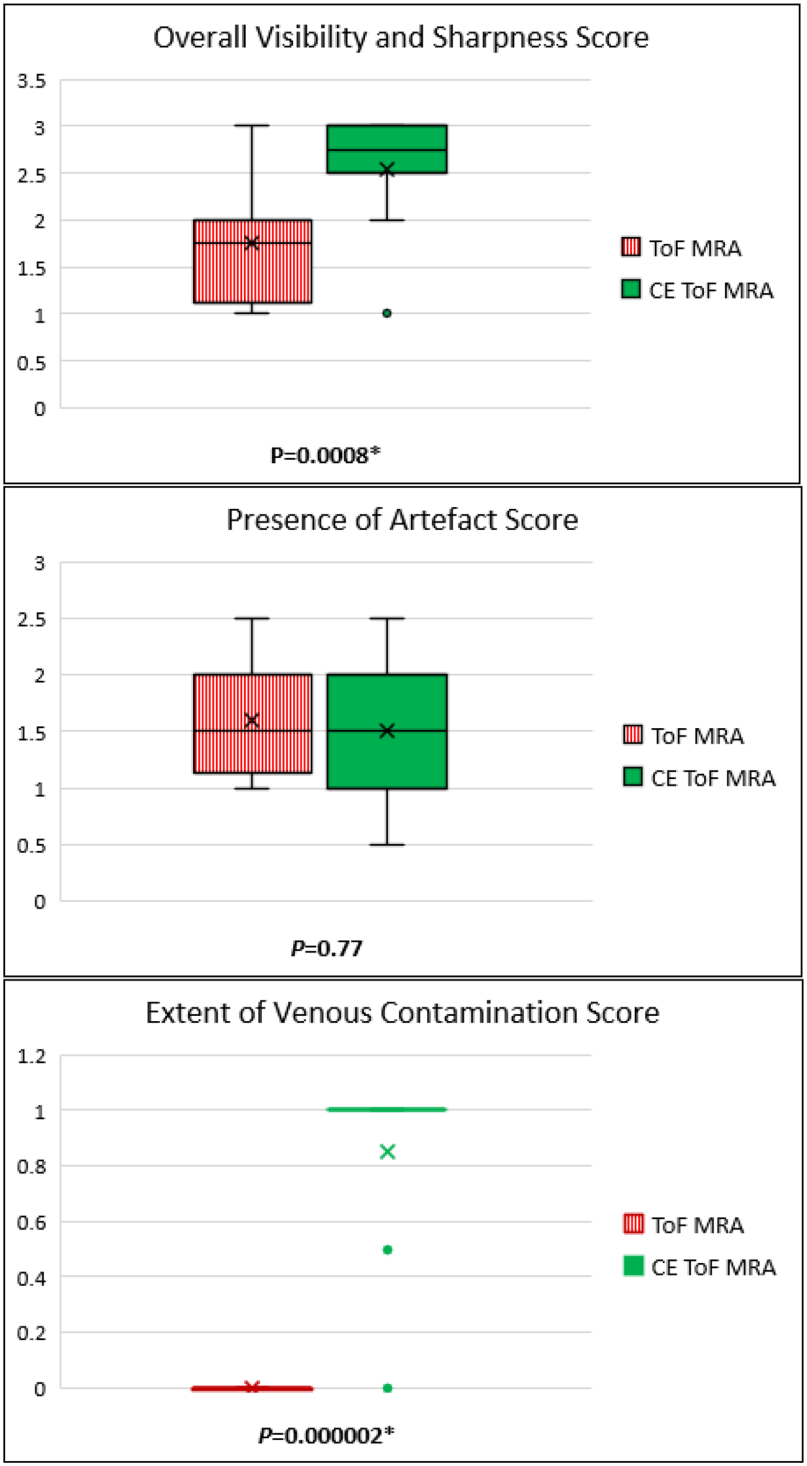
Qualitative analysis comparing standard ToF MRA and CE ToF MRA with boxplots and the Wilcoxon signed-rank test. For Overall Visibility and Sharpness Score, there was a statistically significant difference in favour of CE ToF MRA over standard ToF MRA. For Presence of Artefacts Score, there was no difference between standard ToF MRA and CE ToF MRA. For Extent of Venous Contamination Score, there was a difference in favour of standard ToF MRA over CE ToF MRA, however this was mild. Box plot shows X (the mean values); the 25th to 75th percentiles; the whiskers (the 5th and 95th percentiles); and the green dots (the outliers). *Statistical significance reached (*P* < 0.05). ToF, Time-of-Flight; MRA, Magnetic Resonance Angiography; CE, Contrast-Enhanced.

A summary of the qualitative analysis is shown in Fig. 1.

**Figure 2:**
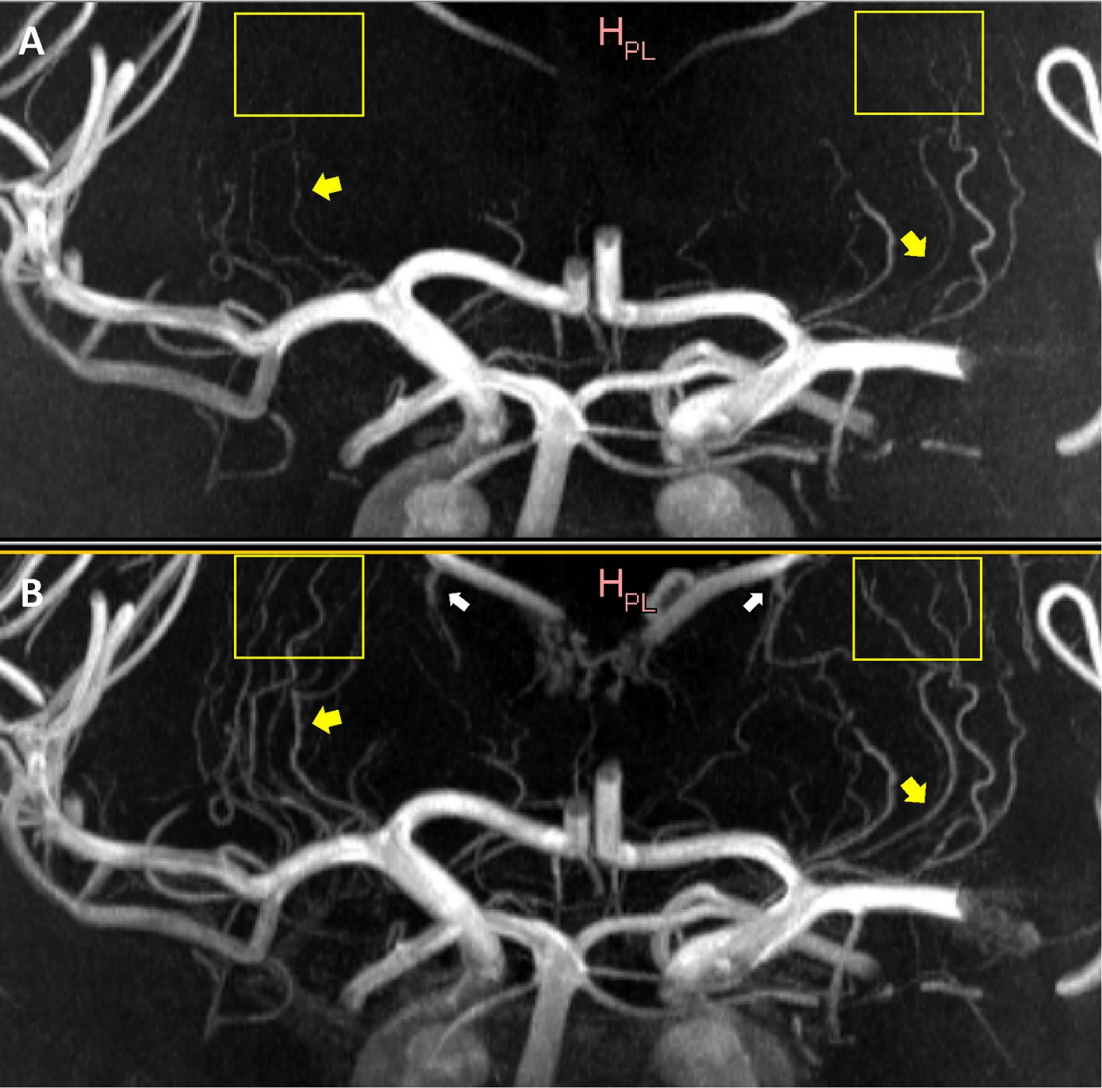
Coronal plane Maximum Intensity Projections comparing standard ToF MRA (**A**) and CE TOF MRA (**B**) for overall visibility and sharpness. The improved clarity of the LSAs in the CE ToF MRA and improved signal in areas of signal loss (yellow arrows) in the standard ToF MRA is shown. The LSAs could also be followed over a longer trajectory (yellow boxes). Also, prominence of the venous system (Choroidal and Thalamostriate Veins draining into the Internal Cerebral Veins; white arrows) is noted in the CE ToF MRA. For Overall Visibility and Sharpness Score scale, the standard ToF MRA was rated 2 (well-defined LSAs) while the CE ToF MRA was rated 3 (excellent definition of the LSAs). ToF, Time-of-Flight; MRA, Magnetic Resonance Angiography; CE, Contrast-Enhanced; LSAs, Lenticulostriate Arteries; MCA, Middle Cerebral Artery.

Overall Visibility and Sharpness Score was higher for CE ToF MRA over standard ToF MRA (2.55 ± 0.64 vs 1.75 ± 0.68; *P* = 0.0008). An illustration of the improved visualisation seen with CE ToF MRA is shown in Fig. 2.

**Figure 3:**
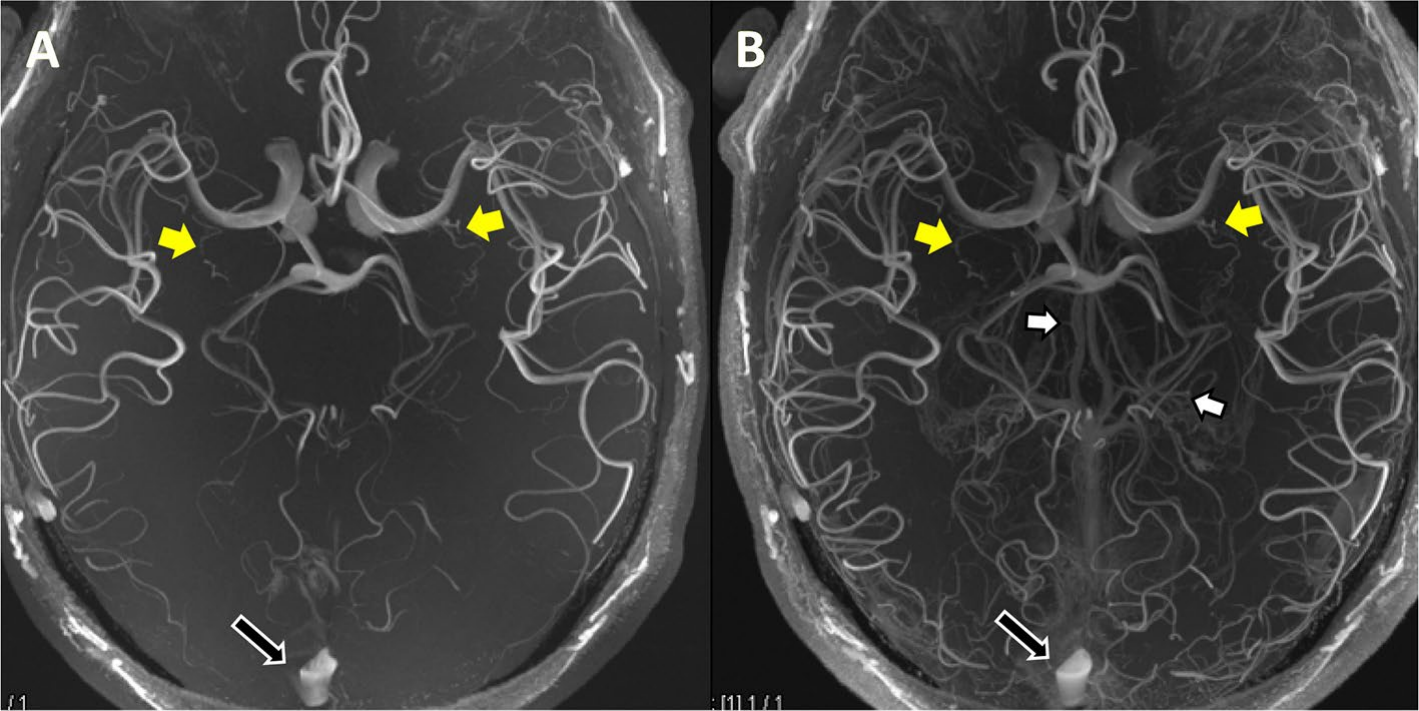
Axial plane Maximum Intensity Projections comparing standard ToF MRA (**A**) and CE ToF MRA (**B**) for extent of venous contamination. The prominence of the internal Cerebral Veins centrally and its tributaries (white arrows) and the Superior Sagittal Sinus (black arrows with white borders) which do not affect the visualisation of the LSAs at the MCA (yellow arrows) is shown. For Extent of Venous Contamination Score scale, this was rated 0 (no venous contamination hampering visibility) in both images. ToF, Time-of-Flight; MRA, Magnetic Resonance Angiography; CE, Contrast-Enhanced; LSAs, Lenticulostriate Arteries; MCA, Middle Cerebral Artery.

For Presence of Artefacts Score, there was no difference (standard ToF MRA 1.6 ± 0.52 vs CE ToF MRA 1.5 ± 0.62; *P* = 0.77). Venous Contamination Score was higher for CE ToF MRA over standard ToF MRA (1 vs 0; *P* = 0.000002) as illustrated in Fig. 3.

### Length and number of LSAs visualised

The mean length of LSA visualised was significantly longer with CE ToF MRA compared to standard ToF MRA (24.4 ± 4.5 vs. 21.9 ± 4.0 mm, *P* = 0.01) as shown in Table 3. A figure format of the length measurements is shown in Supplementary Fig. 2. There was no significant difference in the number of visible LSAs (4.6 ± 2.1 vs. 4.9 ± 2.3, *P* = 0.19, Table 3).

**Table 3:** Quantitative Analysis comparing standard ToF MRA, and CE ToF MRA based on Signal Intensities, Signal to Noise Ratio, Contrast to Nosie Ratio, number of visible LSAs and Straight Length of the LSAs.

Quantitative analysis parameters	Standard ToF MRA	CE ToF MRA	*P* value
LSA Signal Intensity	239.4 ± 97.5	262.1 ± 98.1	0.02*
Brain Signal intensity	62.3 ± 6.0	65.5 ± 5.9	0.01*
Background Noise standard deviation	4.3 ± 0.4	4.5 ± 0.5	0.08
SNR_LSA_	38.9 ± 16.1	40.7 ± 15.2	0.08
SNR_BRAIN_	10 ± 0.5	10.1 ± 0.7	0.77
CNR_LSA-BRAIN_	28.9 ± 16.0	30.6 ± 14.0	0.08
Number of visualised LSAs	4.6 ± 2.1	4.9 ± 2.3	0.19
Straight Length of LSA (mm)	21.9 ± 4.0	24.4 ± 4.5	0.01*

All values expressed as Mean ± SD (standard deviation). ToF, Time-of-Flight; MRA, Magnetic Resonance Angiography; CE, Contrast-Enhanced. LSA: Lenticulostriate Artery; SNR, Signal to Noise Ratio; CNR, Contrast to Nosie Ratio; mm, milimeters. *Statistical significance reached (*P* < 0.05).

### Signal and contrast to noise ratios

The signal intensity of blood in the LSAs was higher with CE ToF MRA compared to standard ToF MRA (*P* = 0.02, Table 3). However, the brain signal intensity was also significantly higher with the CE ToF MRA (*P* = 0.01). This overall resulted in an increased CNR for the CE ToF MRA sequence, though there was no significant difference in the CNR for CE ToF MRA and standard ToF MRA (30.6 ± 14.0 vs. 28.9 ± 16.0, *P* = 0.08).

## Discussion

Our prospective study in patients with symptomatic lacunar stroke demonstrates that CE ToF MRA results in improved visualisation of the small cerebral perforating arteries compared with standard ToF MRA at 7 T. The use of contrast was associated with improved visualisation and clarity of the lenticulostriate perforating arteries on our visual rating scale, and a greater visible length of the perforators. The improvement visualisation noted in our study, did not correspond with one previous study^13^; this could be due to fewer subjects having pre-contrast ToF MRA were available for comparison^13^. The greater visible length on the other hand, corresponds to findings in earlier studies^13,21^. Use of contrast did not show a significant increase the number of perforators that were visible, similar to another study^13^. On quantitative analysis, CE ToF MRA was associated with increased signal in the small perforating arteries although the increase in contrast to noise ratio was not significant. This could be explained by the increase in the background noise and brain tissue signal in the CE ToF MRA sequence. Another concern with the use of contrast is that it could increase venous contamination which would impair visualisation of the small perforating vessels^13,22^. However, although venous contamination was slightly increased, this did not affect perforator artery visualisation. Therefore, our results suggest that contrast enhancement offers improvements in imaging of the small cerebral perforating arteries in this patient cohort.

It is important to note that we used a TR of 13 ms. This was chosen after a validation study on our scanner, using non-contrast ToF MRA sequences at TRs of 12 ms, 13 ms, and 24 ms, which showed good vessel visualisation at a TR of 13 ms^23^. While some 7 T studies have used a TR of 25 to 33 ms,^24,25^ other studies have used shorter TRs of 15 to 16 ms^13,17,26^. Our chosen shorter TR enabled us to achieve acquisition times acceptable for scanning patient populations (less than 10 min) while obtaining a coverage of 156 mm with high resolution of 0.24 × 0.24 × 0.32 mm^3^. Although our acquisition time is comparable to other studies with an average of 9 to 11 min^13,17,22,26^, subject motion is expected with prolonged imaging sequences and poses a challenge for obtaining high quality images. Application of methods for motion correction either prospectively^27^ or retrospectively^28^ may be useful in improving the visualisation of these small perforating arteries.

Our study has a number of strengths. It was a prospective study with a predefined imaging protocol. All patients were imaged using an identical scanner and imaging characteristics, and standard ToF MRA and CE TOF MRA sequences were performed sequentially during the same imaging sessions and comparison of both sequences were made in the same participants at the same time. The two imaging sequences were assessed using a number of different analysis techniques which showed broadly consistent results. Visual rating was carried out by two experts blinded to each other’s results.

Our study, however, also has some limitations. The sample size was small, although by directly comparing results within the same patient between the two sequences, this was sufficient to show significant differences. We limited our study to the perforators arising from the MCA and ACA. The goal was to assess the LSAs, which most often originate from the proximal (M1) segment of the MCA^29^ as well as the proximal (A1) segment of the ACA^30^. We used a narrow FOV with an acquisition time of approximately 10 min, making it difficult to include the whole Circle of Willis or other perforating arteries, such as the thalamoperforator arteries arising from the proximal posterior cerebral artery, posterior communicating artery and the tip of the basilar artery. In some cases, some LSAs could not be tracked beyond the area of coverage. Enlarging the FOV would invariably lead to increased acquisition time, prolonging the time a participant spends lying still in the scanner^31^. A method of accelerating image acquisition without affecting image quality like compressed sensing^32,33^ has not been evaluated for CE ToF MRA; this is an area for further research. Thirdly, the use of contrast does have some potential side effects. Concerns in relation to nephrotoxicity, the development of a very rare condition of nephrogenic systemic fibrosis (NSF), and the potential impact of long-term gadolinium retention, particularly in the brain, have been raised^34^. For this reason, creatinine clearance was checked in all patients before imaging and any participants with impaired renal function were excluded. Also, as a precaution, we used a non-ionic chelate gadolinium-based contrast agent (low NSF risk category) and followed our local standard operating procedure to mitigate against this risk.

## Conclusion

Currently, 7 T MRI is largely used as a research technique, where it provides a unique opportunity to visualise the perforating arteries and their pathology. In such studies, the use of contrast enhancement improves visualisation of the LSAs, and its use may aid in detecting pathology in cSVD, and in studying the effects of therapeutic interventions.

## Supplementary Information

Supplementary Information.

## Data Availability

The data that support the findings of this study are available from the corresponding author, although restrictions apply to the availability of these data, and so are not publicly available. Data are however available upon reasonable request subject to permission being obtained from the University of Cambridge.

## Abbreviations

7 T: 7 Tesla
CE: Contrast-enhanced
cSVD: Cerebral small vessel disease
FOV: Field-of-view
LSAs: Lenticulostriate arteries
NSF: Nephrogenic systemic fibrosis
ToF MRA: Time-of-flight magnetic resonance angiography
TR: Repetition time
