# Supplementary material for: Visualisation of lenticulostriate arteries using contrast-enhanced time-of-flight magnetic resonance angiography at 7 Tesla
Source: Sci Rep. 2022 Nov 24;12:20306. doi: 10.1038/s41598-022-24832-z (PMC9700841; doi:10.1038/s41598-022-24832-z)
Supplement: Supplementary file 1 — Supplementary Information. [file 41598_2022_24832_MOESM1_ESM.docx]

**Supplementary Materials**

**Supplementary Figure S1:** Axial plane image illustrating placement of ROIs for measurement of signal intensities of blood in the LSA (a, mean, 189.8), signal intensity of brain (b, mean, 72) and background noise standard deviation (c, StDev 4.2).

***
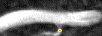
****
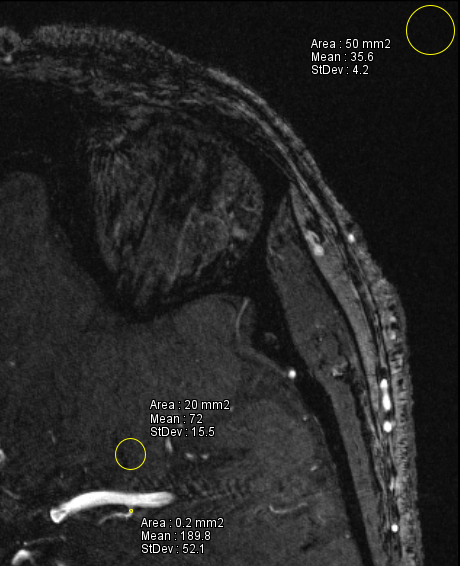
*

**c**

**b**

**a**

Inset (a): Magnification of ROI placement at the LSA*.* ROI area was 0.2mm^2^ for the LSA and 20mm^2^ for brain and 50mm^2^ for background noise. ROI, Region of Interest; LSA, Lenticulostriate artery; StDev, Standard Deviation.

**Supplementary Figure S2:** Coronal plane Maximum Intensity Projection illustrating measurement of the length of the most lateral LSA as a straight (through-space) linear distance in Standard ToF MRA (A) and CE TOF MRA (B).


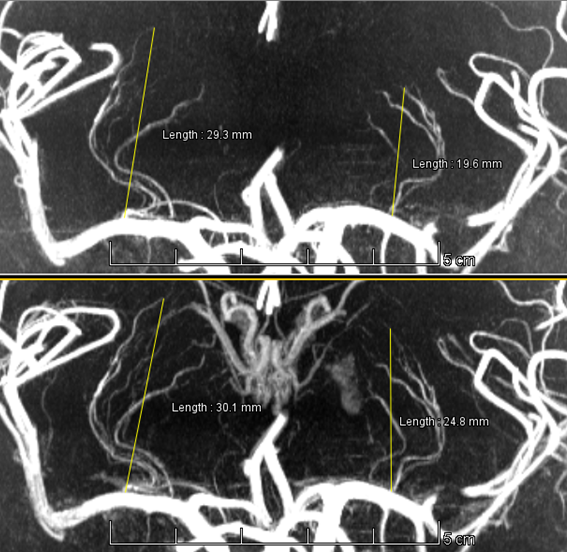


**B**

**A**

The LSAs in the CE TOF MRA on the right (30.1mm) and left (24.8mm) could be followed over a longer distance compared to those on the Standard ToF MRA on the right and left (29.3mm and 19.6mm respectively). ToF, Time-of-Flight; MRA, Magnetic Resonance Angiography; CE, Contrast-Enhanced; LSAs, Lenticulostriate Arteries.
